# Don't Lose Your Cool With Cryotherapy: The Application of Phase Change Material for Prolonged Cooling in Athletic Recovery and Beyond

**DOI:** 10.3389/fspor.2020.00118

**Published:** 2020-10-15

**Authors:** Susan Y. Kwiecien, Malachy P. McHugh, Glyn Howatson

**Affiliations:** ^1^Nicholas Institute of Sports Medicine and Athletic Trauma, Lenox Hill Hospital, New York, NY, United States; ^2^Department of Sport, Exercise and Rehabilitation, Northumbria University, Newcastle upon Tyne, United Kingdom; ^3^Water Research Group, North West University, Potchefstroom, South Africa

**Keywords:** muscle damage, injury, cooling, recovery modalities, recovery strategy

## Abstract

Strenuous exercise can result in muscle damage in both recreational and elite athletes, and is accompanied by strength loss, and increases in soreness, oxidative stress, and inflammation. If the aforementioned signs and symptoms associated with exercise-induced muscle damage are excessive or unabated, the recovery process becomes prolonged and can result in performance decrements; consequently, there has been a great deal of research focussing on accelerating recovery following exercise. A popular recovery modality is cryotherapy which results in a reduction of tissue temperature by the withdrawal of heat from the body. Cryotherapy is advantageous because of its ability to reduce tissue temperature at the site of muscle damage. However, there are logistical limitations to traditional cryotherapy modalities, such as cold-water immersion or whole-body cryotherapy, because they are limited by the duration for which they can be administered in a single dose. Phase change material (PCM) at a temperature of 15°C can deliver a single dose of cooling for a prolonged duration in a practical, efficacious, and safe way; hence overcoming the limitations of traditional cryotherapy modalities. Recently, 15°C PCM has been locally administered following isolated eccentric exercise, a soccer match, and baseball pitching, for durations of 3–6 h with no adverse effects. These data showed that using 15°C PCM to prolong the duration of cooling successfully reduced strength loss and soreness following exercise. Extending the positive effects associated with cryotherapy by prolonging the duration of cooling can enhance recovery following exercise and give athletes a competitive advantage.

## Rationale

Exercise often involves high intensity physical and physiological stress that ultimately results in structural damage to the skeletal muscle (Armstrong, 1990; Armstrong et al., 1991; Clarkson and Sayers, 1999; Proske and Morgan, 2001; Proske and Allen, 2005). Three pathways exist during exercise that result in the structural damage of the muscle. These include: the increase in muscle temperature resulting from exercise-induced heat generation (Arbogast and Reid, 2004), the metabolic stress that commonly occurs during exercise of high intensity or prolonged durations (Spiteller, 2006; Clanton, 2007; Supinski and Callahan, 2007; Tee et al., 2007), and/or the direct mechanical stress to the muscle (Staublr, 1989; Friden and Lieber, 1992, 2001; Proske and Morgan, 2001; Lieber, 2018). The initial structural damage occurring within the muscle fiber initiates a positive feedback mechanism during which the aforementioned initial damage response is exacerbated (Kendall and Eston, 2002; Merrick, 2002; Howatson and van Someren, 2008). This phase of muscle damage is referred to as the secondary damage response. Ultimately, secondary muscle damage compounds the symptoms of exercise-induced muscle damage and results in impaired muscle function in the hours and days following exercise (Lapointe et al., 2002). If not managed correctly, these effects can be detrimental to an athlete's recovery and subsequent performance. As a result, accelerating recovery following strenuous exercise has been the focus of much research; particularly when there is inadequate recovery between repeat exercise exposures. In these scenarios, rapid deployment of a recovery strategy is important for athletes to accelerate the return to optimal performance.

Exposure of the damaged muscle to cold (cryotherapy) is believed to retard the secondary injury process (Merrick et al., 1999; Merrick, 2002). Cryotherapy, the reduction of tissue temperature by the withdrawal of heat from the body (Michlovitz, 1990), refers to a range of cooling modalities such as local ice application to the skin (Yackzan et al., 1984; Gulick et al., 1996; Oakley et al., 2013; Nogueira et al., 2019), cold water immersion (CWI) of a large part of the body (Lane and Wenger, 2004; Yeargin et al., 2006; Vaile et al., 2007, 2008, 2010; Halson et al., 2008; Montgomery et al., 2008; Peiffer et al., 2008; Rowsell et al., 2009, 2011; Brophy-Williams et al., 2011; Bleakley et al., 2012; Leeder et al., 2012, 2019; Versey et al., 2013; Webb et al., 2013; Roberts et al., 2015; Garcia et al., 2016; Machado et al., 2016; Wilson et al., 2018), whole body cryotherapy (Banfi et al., 2009, 2010; Costello et al., 2011, 2015; Hausswirth et al., 2011; Pournot et al., 2011; Ziemann et al., 2012; Fonda and Sarabon, 2013; Guilhem et al., 2013; Bleakley et al., 2014; Ferreira-Junior et al., 2015; Vieira et al., 2015; Rose et al., 2017; Broatch et al., 2019; Krueger et al., 2019; WBC) and more recently phase change material (PCM) cooling (Clifford et al., 2018; Kwiecien et al., 2018, 2020a,b; Brownstein et al., 2019; Mullaney et al., 2020), that are employed in various contexts. The most popular cryotherapy modality used following exercise is CWI involving immersion of a large surface area of the body, typically immersion of at least the legs up to at least the umbilicus, in cold water. Most commonly CWI occurs in water temperatures of 15°C or less for a single duration of 15 min or less (Leeder et al., 2012). Evidence supports the use of CWI for accelerating recovery of soreness (Barnett, 2006; Bailey et al., 2007; Montgomery et al., 2008; Ingram et al., 2009; Pournot et al., 2010; Hausswirth and Le Meur, 2011; Pointon et al., 2011; Bleakley et al., 2012; Leeder et al., 2012; Minett et al., 2012; Pointon and Duffield, 2012; Elias et al., 2013; Poppendieck et al., 2013; Hohenauer et al., 2015; Machado et al., 2015; Ihsan et al., 2016; Siqueira et al., 2018). There is also some evidence to support the use of CWI for accelerating recovery of blood markers of muscle damage (Leeder et al., 2012; Hohenauer et al., 2015; Siqueira et al., 2016; Dupuy et al., 2018) and inflammation (Vieira Ramos et al., 2016; Dupuy et al., 2018), as well as functional recovery (Vaile et al., 2008; Leeder et al., 2019) following exercise. However, evidence to support its use for accelerating recovery of strength loss following exercise remains equivocal (Bleakley et al., 2012; Leeder et al., 2012; Poppendieck et al., 2013; Versey et al., 2013; Hohenauer et al., 2015; Machado et al., 2015). Comparably, some studies suggest that WBC might be beneficial in accelerating subjective recovery of soreness (Banfi et al., 2010; Hausswirth et al., 2011; Pournot et al., 2011; Ziemann et al., 2012; Fonda and Sarabon, 2013; Bleakley et al., 2014; Costello et al., 2015; Rose et al., 2017), strength loss (Hausswirth et al., 2011), and might mitigate the signs of functional overreaching (Schaal et al., 2015). However, more recently, there remains little evidence to support improvements in functional recovery (Bleakley et al., 2014; Lombardi et al., 2017; Rose et al., 2017; Broatch et al., 2019; Krueger et al., 2019). On the contrary, local ice application does not improve the symptoms associated with soreness or strength loss (Nogueira et al., 2019). Thus, local ice application is generally not effective in the treatment of structural damage following exercise. Ultimately, the lack of evidence identifying specific guidelines concerning traditional cryotherapy treatment application, temperature, duration, and frequency, as well as the variability in exercise models utilized throughout the literature, likely contribute to the controversy surrounding the efficacy and practicality of cryotherapy for accelerating recovery following exercise. As a result, no consensus exists for optimal cryotherapy treatment criteria and there remains a large gap in the scientific basis for administering cryotherapy for anything other than subjective recovery following exercise.

Recent evidence suggests that the physiological changes that occur following cryotherapy are primarily dependent on the reduction in intramuscular temperature (Wilcock et al., 2006; White and Wells, 2013; Ihsan et al., 2016) and only secondarily reliant on vasoconstriction leading to a decrease in blood flow (Gregson et al., 2011; Ihsan et al., 2013; Mawhinney et al., 2013, 2020) which might decrease muscle metabolism and inflammation, resulting in a reduction in the proliferation of secondary damage (Meeusen and Lievens, 1986; Knight, 1995; Eston and Peters, 1999; Merrick et al., 1999; Merrick and McBrier, 2010). Evidence from animal models suggests that the optimal muscle temperature range for reducing cellular metabolic activity (Osterman et al., 1984; Sapega et al., 1988) and oxygen demand (Fuhrman, 1959; Fuhrman et al., 1961) without causing tissue damage, is 10–15°C (Sapega et al., 1988). However, *in vivo* intramuscular temperatures below 20°C during traditional cryotherapy application in humans have not been reported (Bleakley and Hopkins, 2010; Bleakley et al., 2012). In order to sustain a clinically relevant reduction in intramuscular temperature, the duration of cryotherapy would have to be prolonged (Peiffer et al., 2009). However, treatment duration does not commonly exceed 30 min because extending the duration of cryotherapy is likely to result in increased discomfort particularly at lower temperatures (Bailey et al., 2007; Vaile et al., 2008; Heyman et al., 2009; Versey et al., 2011) or can be unsafe (Tipton et al., 2017). Rapid reductions in skin temperature, before muscle and core temperatures can catch up, might result in cold related injury to the skin (Gage, 1979; Wilke and Weiner, 2003; Selfe et al., 2007) because the skin is most prone to irreversible damage. To date, maintaining a reduction in muscle temperature without causing cold related injury to the skin could only be achieved by administering traditional cryotherapy modalities (ice, gel packs, CWI, WBC, etc.) in an intermittent fashion (Mac Auley, 2001). However, Cheng et al. (2017) recently reported intramuscular temperature reductions to approximately 15°C following a 120-min localized cooling intervention administered to the upper arm, making the case for a prolonged duration of cryotherapy application in order to achieve clinically relevant reductions in intramuscular temperature. Nevertheless, the protocol utilized by Cheng et al. (2017), 120 min of cooling with ice-chilled water-perfused arm cuffs, is atypical from common practice in the application to athletes in the “real world” (Ihsan et al., 2020).

**Figure 1:**
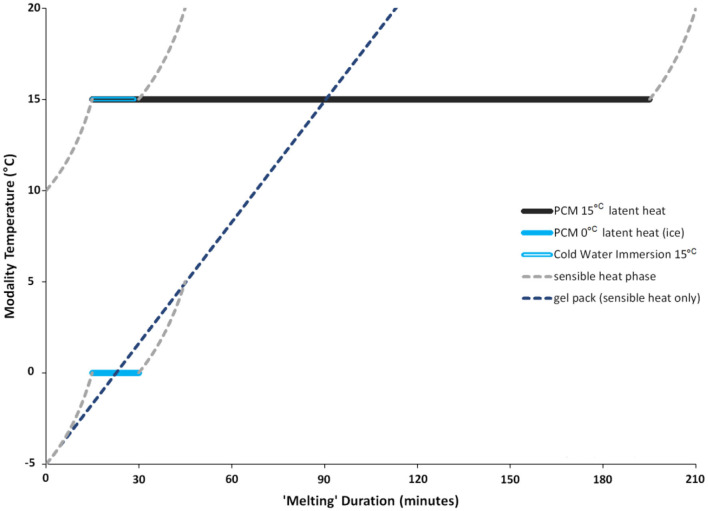
Sample melting pattern of PCM with a phase transition point of 15°C, or 0°C for comparison, and for material with only sensible heat properties (e.g., ice pack). Latent heat period (solid line) demonstrates a change in phase while maintaining a constant temperature. The latent heat phase is longer for 15°C PCM than it is for 0°C PCM (ice melting from solid to liquid). The 15°C PCM is compared with CWI that is kept at a constant temperature of 15°C for a 15-min duration. Once the CWI temperature is no longer held at a constant, the water temperature will begin to equilibrate to room temperature (sensible heat). The 0°C PCM (ice) is compared with a gel pack that is initially frozen at −5°C but warms up to 20°C as it equilibrates with room temperature, so it does not change phase and experiences only sensible heat. Note: PCM melting duration is dependent on the temperature gradient between skin and PCM, the PCM phase transition point, the area covered by PCM and the volume of the PCM.

In humans, the magnitude of change in tissue temperature has been positively correlated with cryotherapy methods that undergo a phase change (Merrick et al., 2003; Dykstra et al., 2009). Specifically, Merrick et al. (2003) demonstrated that modalities such as ice that change phase while they melt (e.g., from solid to a liquid) cause lower skin and intramuscular temperatures than cryotherapy modalities such as gel packs that do not possess these properties (ice bag: 6.5°C, skin temperature; 27.8°C, 1 cm intramuscular temperature; vs. gel pack: 9.9°C, skin temperature; 29.5°C, 1 cm intramuscular temperature; Merrick et al., 2003). A phase change is important because it greatly enhances the ability of a cryotherapy modality to absorb heat (Merrick et al., 2003). The phase change relates to a property called “enthalpy of fusion,” which is the quantity of heat required to make the material change phase (Merrick et al., 2003). Enthalpy of fusion greatly enhances the ability of a cold modality to absorb heat by prolonging the latent phase, and thus results in a greater ability to reduce intramuscular temperature. When a substance is changing phases, there is only a change in phase but no change in temperature. This “hidden” energy is defined as the latent phase. On the contrary, sensible heat is heat that can be felt and measured by a thermometer. Neither gel packs nor WBC undergo a phase change, meaning that both modalities only experience sensible heat loss as their temperature equilibrates with the ambient temperature (Figure 1). Similarly, CWI does not change phase, but its temperature can be artificially maintained at a constant creating an artificial latent phase period. On the contrary, when ice is heated by exposure to the human body, its temperature increases, and it experiences a change in phase as it melts. While undergoing the phase change, ice experiences latent heat loss during which the temperature remains constant (Figure 1). Therefore, modalities experiencing the latent phase have an advantage over modalities only capable of experiencing a sensible heat phase, by providing a greater cooling potential.

Although ice is the most commonly utilized PCM for exercise recovery, its latent phase of 0°C limits it from maintaining a cooling capacity for prolonged periods. Luckily, the latent phase of any PCM can be manipulated. The duration of the latent phase can be prolonged as the temperature of phase change increases above 0°C. The cooling effect of any PCM is dependent on the capacity to absorb heat during periods when external heat load or body heat production exceeds heat loss. Therefore, the duration of the latent phase is variable and dependent on the temperature gradient between the skin and the PCM, the PCM phase transition point, the area covered by PCM and the volume of the PCM (Tiest et al., 2012; Hassabo, 2014). For example, PCMs will melt faster if the skin is warmer, PCM with a phase transition point of 10°C will not hold that temperature as long as PCMs with a set point of 15°C, and a small volume of PCM will melt faster than a larger volume.

**Figure 2:**
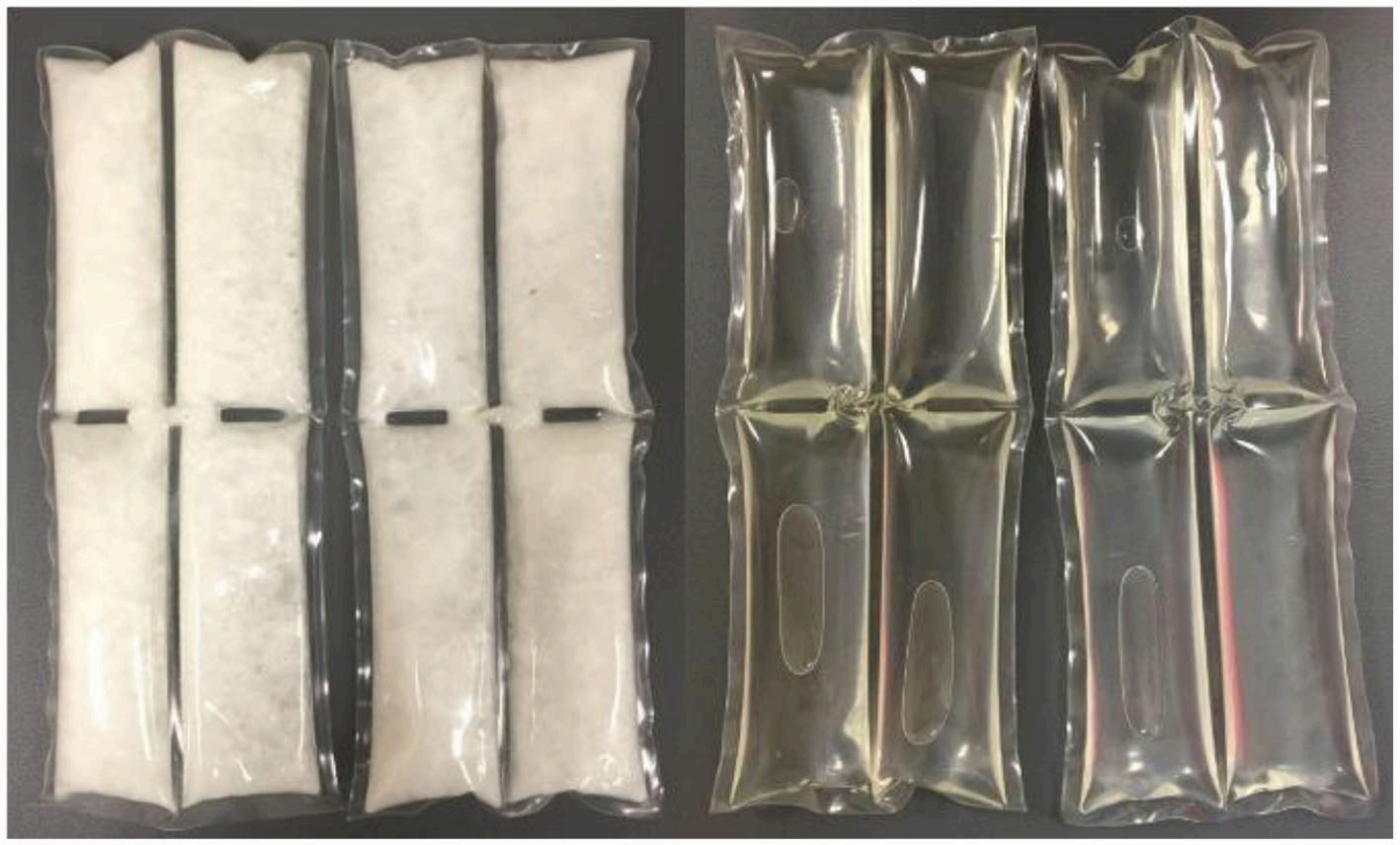
Two Glacier Tek 15°C PCM packs in the frozen state **(left)**, and in their melted state **(right)**.

The 15°C PCM packs used throughout the course of the subsequently described research studies are filled with a proprietary blend of fully hydrogenated natural fats certified by the U.S. Food and Drug Administration as food-grade chemicals such as palm oil, palm kernel oil, rapeseed oil, coconut oil and soybean oil, mixed with sodium chloride, and encapsulated in flexible plastic (Glacier Tek USDA BioPreferred PureTemp PCM, Plymouth, MN, USA). Similar to ice, the 15°C PCM are firm and look like wax when in their frozen solid state and look like vegetable oil once they reach their melted liquid state (Figure 2). A PCM with a latent phase of 15°C is capable of safely prolonging and maintaining the duration of cooling for 3 h (Kwiecien et al., 2019), while avoiding the need for repeat applications. If there is a need to extend the duration of application beyond 3 h, a fresh set of “frozen” PCM packs can be administered for an additional 3 h. Applying 15°C PCM packs directly to the skin fitted under a garment might be less time consuming, logistically simpler to implement than other cryotherapy modalities, and is more practical particularly because they can simultaneously deliver a cooling treatment while the individual continues activities of daily living. Furthermore, 15°C PCM packs can be applied concurrently to multiple athletes, or even entire teams at a time. Recent evidence suggests that prolonging the duration of cooling for three (Clifford et al., 2018; Brownstein et al., 2019; Mullaney et al., 2020) to 6 h (Kwiecien et al., 2018, 2020a) is well tolerated by recreational and professional athletes alike and can accelerate more than just subjective recovery following exercise. Therefore, this narrative review will aim to summarize the current evidence in support of prolonging the duration of cooling by using 15°C PCM, for its capacity to accelerate and aid recovery following exercise. As the literature has already summarized the mechanisms of cryotherapy and its effects on the mechanisms involved with exercise-induced muscle damage (Versey et al., 2013; Hohenauer et al., 2015; Machado et al., 2015; Ihsan et al., 2016; Dupuy et al., 2018; Stephens et al., 2018), an extensive summary is beyond the scope of this review.

## Evidence

Much of the previous research utilizing PCM cooling at phase transition points ranging between 10 and 31°C has focused on its temperature-regulating effect (Gao et al., 2010, 2011), and the ability of PCM to elicit thermal comfort from heat strain occurring during strenuous activity (Bennett et al., 1995; Zhang, 2003; Chou et al., 2008; Kenny et al., 2011; House et al., 2013), between two bouts of exercise (Duffield and Marino, 2007; Hausswirth et al., 2012), or following exercise (Purvis and Cable, 2000; Tate et al., 2008; Barwood et al., 2009; Brade et al., 2010). In this context, most of the research has applied PCM through pockets in vests worn on the chest in an attempt to reduce elevations in core temperature, for treatment durations longer than CWI. Following strenuous activity, 24°C PCM vests have been shown to be more effective at cooling the skin than 28°C vests, but neither had an effect on core temperature (Gao et al., 2011). A PCM with a melting point <24°C might reduce core temperature more effectively (Gao et al., 2011). In rested individuals, local application of 15°C PCM to the quadriceps for 3 h reduced core temperature by 0.28°C compared with a 0.25°C reduction from 15 min of 15°C CWI (Kwiecien et al., 2019). Although the difference in the reduction in core temperature from both PCM and CWI was negligible, the CWI reduced core temperature faster than the PCM treatment but the reduction in core temperature was maintained for the 3-h duration of 15°C PCM application (Kwiecien et al., 2019). In comparison, following exercise, a pooled data analysis of 13 studies found that CWI reduced core temperature by 0.84°C (Stephens et al., 2018). Following exercise, cryotherapy is expected to induce a greater magnitude in the reduction of core temperature than at rest due to the exercise-induced hyperthermia resulting in a larger thermal gradient. Unfortunately, the effects of 15°C PCM cooling on core and muscle temperature remain to be elucidated following exercise.

In order to induce reductions in local metabolic demand (Ho et al., 1995; Merrick, 2002; Schaser et al., 2006) and the inflammatory response that occurs after the acute structural trauma at the site of injury or muscle damage (Ciolek, 1985; Knight, 1985, 1995; Merrick et al., 1999; Merrick, 2002; Schaser et al., 2006; Swenson et al., 2007), intramuscular temperature must drop to sufficiently low levels (10–15°C; Sapega et al., 1988). Since the secondary injury response extends for several hours post-exercise, a single 15-min CWI treatment may be an inadequate dose to influence recovery. Furthermore, extending the duration of a single CWI treatment would not be well tolerated. As a result, previously the only way to sustain a reduction in intramuscular temperature without causing cold related injury to the skin was through repeat applications of traditional cryotherapy modalities at frequent intervals. However, this practice is not commonplace amongst athletes, as they are unlikely to comply with such a demanding treatment schedule because it would mean that athletes must remain on site for an extended period, and there are major logistical challenges to treating entire teams at one time. As a result, the duration for which muscle temperature is reduced during traditional cryotherapy treatment is likely too short to elicit meaningful reductions in muscle temperature for durations long enough to be clinically relevant (10–15°C; Sapega et al., 1988). Although the intramuscular temperatures reported from 3 h of 15°C PCM cooling are nowhere close to those necessary for reducing local muscle metabolism (26.0 ± 2.2°C at 1 cm and 28.2 ± 2.8°C at 3 cm of the vastus lateralis), the reduction was safely maintained for a prolonged and continuous duration in individuals at rest (Kwiecien et al., 2019). Furthermore, these temperatures were comparable to those occurring from 15 min of 15°C CWI even though two PCM packs (864 cm^2^ area; 32.4 × 2 × 13.3 cm) were administered locally and fitted directly on the skin over the quadriceps of each leg, while CWI treatment involved whole body immersion up to the umbilicus (Kwiecien et al., 2019). Therefore, prolonging the treatment duration using 15°C PCM cooling affords athletes the opportunity to safely receive cryotherapy treatment for an extended duration with the ability to leave the training facility and resume their normal routines.

Recent studies examining the effects of prolonged 15°C PCM cooling on indices of recovery in untrained (Kwiecien et al., 2018) and trained (Kwiecien et al., 2020a) individuals found that 6 h of PCM cooling not only accelerated recovery of soreness, but also accelerated recovery of strength loss on the days after isolated eccentric quadriceps exercise (Table 1). Importantly, the protection provided by the PCM cooling after an initial bout of eccentric exercise did not interfere with the repeated bout effect, whereby an initial bout confers an adaptive protective effect for a subsequent bout of damaging exercise (Kwiecien et al., 2020a). Similarly, 3 h of 15°C PCM cooling has also been shown to accelerate recovery of quadriceps strength following soccer match play (Clifford et al., 2018; Brownstein et al., 2019; Table 1) and shoulder internal rotation strength and grip strength following baseball pitching (Mullaney et al., 2020; Table 1). Alleviating strength loss at a rate faster than normal could allow athletes to be better prepared for subsequent performance, giving them a competitive advantage over the opposition. The beneficial effects of PCM cooling on recovery of strength loss following exercise in these five studies are in contrast to the results of research utilizing CWI for recovery, which has generally shown little or no benefit for recovery of strength loss following exercise (Leeder et al., 2012; White and Wells, 2013; Ihsan et al., 2016). Overall, these results suggest that prolonging the duration of cryotherapy might successfully reduce the proliferation of secondary muscle damage and decrease the magnitude of repair necessary to achieve pre-exercise functional integrity, thereby shortening the time required to attain full recovery of muscle strength.

**Table 1:** Summary of evidence available on PCM studies applying 15°C PCM cooling for post-exercise recovery.

**Study**	**Population**	**Exercise type**	**Cooling technique**	**Cooling duration**	**Variables reported**	**Main outcomes**
Kwiecien et al. (2018)	Recreational athletes	Isolated eccentric exercise of the quadriceps	1) Direct PCM cooling 2) Indirect PCM cooling 3) Control	6 h on the quadriceps	↓ Skin temperature ↓ Strength loss ↓ Soreness	• 6 h of direct local PCM cooling was well tolerated • Recovery of strength loss and soreness was accelerated • Leg receiving indirect cooling was not statistically different from direct cooling indicating a potential systemic effect
Clifford et al. (2018)	Professional athletes	Soccer match	1) Direct PCM cooling 2) Control	3 h on the quadriceps	↓ Strength loss ↓ Soreness ↑ Counter movement jump (results published in McHugh et al., 2019)	• PCM cooling can provide a practical means of delivering prolonged post-exercise cooling to entire teams of athletes • PCM cooling can accelerate recovery in elite athletes
Brownstein et al. (2019)	Semi-professional athletes	Soccer match	1) Direct PCM cooling 2) Control	3 h on the quadriceps	↓ Strength loss ↓ Voluntary activation ✘ Soreness ✘ Counter movement jump ✘ Reactive strength index	• PCM cooling accelerated recovery of central nervous system function but not muscle contractile function • The lack of effect on measures of physical function or perceptual responses might have been due to the relatively small magnitude of change in most of the outcome measures studied, which could be related to the training status of the study participants
Kwiecien et al. (2020a)	Regularly participating in team-sport or other forms of physical exercise but not eccentrically trained	Isolated eccentric exercise of the quadriceps	3) Direct PCM cooling 4) Control	6 h on the quadriceps	↓ Skin temperature ↓ Strength loss ↓ Soreness ✘ Creatine kinase ✘ High sensitivity c-reactive protein	• Recovery of strength loss and soreness was accelerated • No effect on blood markers of muscle damage • Exercise was repeated but repeated bout effect was not hindered by initial cooling (no strength loss or soreness after second exercise bout)
Kwiecien et al. (2020b)	Recreational athletes	Marathon run	1) Direct PCM cooling 2) Control	3 h on the quadriceps	✘ Strength loss ✘ Soreness ✘ Counter movement jump ✘ Creatine kinase ✘ High sensitivity c-reactive protein	• No effect on accelerating recovery of any variable • Might be related to either shorter duration of cooling or exacerbated damage response following marathon • Soreness was inversely correlated with number of prior marathons
Mullaney et al. (2020)	Collegiate athletes	Baseball pitching	1) Direct PCM cooling 2) Control	3 h on the shoulder and elbow	↓ Strength loss ✘ Soreness ✘ Creatine kinase	• PCM cooling can be applied comfortably to the arm and accelerates recovery of muscle function in baseball pitchers

*↓ = decrease, ↑ = increase, ✘ = no effect*.

While prolonged cooling using 15°C PCM was shown to be successful in accelerating recovery following eccentric exercise (Kwiecien et al., 2018, 2020a), soccer (Clifford et al., 2018; Brownstein et al., 2019), and baseball pitching (Mullaney et al., 2020), no benefit of accelerated recovery from 15°C PCM cooling was found when administered for 3 h in runners following a marathon (Kwiecien et al., 2020b; Table 1). It is possible that the efficacy of prolonged cooling using 15°C PCM is dependent on the mode of exercise stress resulting in muscle damage, i.e., mechanical vs. metabolic stress. Alternatively, the lack of effect on recovery following a marathon could be due to the combination of the long duration of exercise and the delay in application of the PCM packs to the athletes. Marathon finish times averaged more than 4 h, and it was more than an hour after race completion before the PCM cooling packs were applied to the quadriceps (Kwiecien et al., 2020b). Thus, treatment began more than 5 h after the commencement of the exercise stress. By contrast, in the two soccer studies (Clifford et al., 2018; Brownstein et al., 2019), the PCM cooling packs were applied within 30 min of exercise cessation, which would correspond to approximately 2 h after the commencement of exercise. Although evidence supports the delayed use of CWI (administered 3 h post-exercise) for accelerating recovery of inflammation and improving next day performance (Brophy-Williams et al., 2011), the exercise session performed prior to the cryotherapy treatment lasted a total duration of only 24 min. Therefore, although the delayed cryotherapy modality was not administered immediately upon cessation of exercise, the exercise duration was of significantly shorter duration than a marathon run. Furthermore, the inflammatory response in the control group only increased by 4.1% 24 h following the high intensity interval exercise session performed by the trained athletes in Brophy-Williams et al.'s (2011) study while the marathon run induced the inflammatory response by 8.6% (Kwiecien et al., 2020b), indicating a greater magnitude of exercise stress.

Of the few studies that have shown accelerated recovery of strength with a post-exercise CWI treatment, some involved exercise in the heat; which results in increased thermal strain and possible central fatigue (Pointon et al., 2011; Minett et al., 2014). In the aforementioned studies, recovery of strength was concomitant with the acute amelioration of voluntary activation and core temperature. It has been suggested that CWI mediated recovery of strength loss following exercise in the heat might not solely reflect recovery from exercise-induced muscle damage but might also include recovery from central fatigue (Ihsan et al., 2016). Therefore, under a large thermal load, CWI might alleviate some of the exercise-induced cerebral perturbations through its ability to ameliorate both hyperthermia and the subsequent central nervous system mediated fatigue (Ihsan et al., 2016), via removal of the heat load alongside preservation of the peripheral physiological state (Minett and Duffield, 2014). However, the peripheral effect from cryotherapy on exercise-induced muscle damage occurring within the skeletal muscle might still be limited by the short cryotherapy duration that occurs during icing, CWI, or WBC. On the contrary, the local effects on skin and muscle temperature occurring during 15°C PCM cooling (Kwiecien et al., 2019) might be insufficient to influence core temperature when the magnitude and duration of thermal load is elevated, such as following a marathon (Deschenes et al., 1998; Mortensen et al., 2008). However, the prolonged duration of cooling possible when using PCM might be advantageous if the goal is to maximize the duration of tolerable decline in peripheral muscle temperature. Recovery with PCM cooling could give athletes an advantage particularly if the duration of cryotherapy application needs to be extended in a safe way.

## Future Directions

To date, PCM cooling has only been studied in exercise recovery models with benefits shown for reducing strength loss and soreness after isolated eccentric exercise (Kwiecien et al., 2018, 2020a), accelerating recovery in professional (Clifford et al., 2018) and semi-professional soccer players (Brownstein et al., 2019), and improving strength recovery in baseball pitchers (Mullaney et al., 2020). These studies have answered some fundamental questions and provide some good “proof of concept” that continuous and prolonged PCM cooling application can help recovery of muscle function, reduction in soreness, and does not seem to attenuate acute adaptive responses (Kwiecien et al., 2020a). Nevertheless, in the exercise recovery paradigm, many questions remain unanswered, and the application of prolonged PCM cooling for injury management warrants more research.

The pilot study, which established the efficacy of PCM cooling as an alternative cryotherapy intervention for recovery of strength and soreness in the quadriceps on the days following bilateral isolated eccentric exercise, implemented a unilateral treatment design (Kwiecien et al., 2018). Therefore, the study had three treatment groups: direct cooling to one leg, indirect cooling to the leg contralateral to the direct cooling leg, and control (no PCM cooling). The treatment effects for strength and soreness were not different between the direct and indirect cooling conditions. Consequently, the results indicated that a systemic effect might have occurred in the indirect cooling leg, which did not receive PCM cooling, from the leg that did receive direct cooling. These results support the previous evidence that implicates a systemic response from unilateral cryotherapy treatment (Allan et al., 2017). A central effect from local 15°C PCM cooling was since confirmed by the reduction in core temperature and increase in heart rate variability evident during PCM treatment in individuals at rest (Kwiecien et al., 2019). These findings suggested that prolonged PCM cooling delivered a systemic, and not just a local effect; and might explain, in part, the accelerated recovery evident from PCM cooling following eccentric exercise (Kwiecien et al., 2020a), soccer match play (Clifford et al., 2018; Brownstein et al., 2019) and baseball pitching (Mullaney et al., 2020). However, the central effect occurring from PCM cooling remains to be directly elucidated in individuals following exercise.

Since PCM cooling can provide prolonged cooling to soft tissues (Kwiecien et al., 2019), there are obvious clinical applications. PCM cooling has the potential to accelerate recovery following muscle injury but this effect has not been examined. In the first 24–48 h after a muscle strain injury or a muscle contusion there is a proliferation of the tissue disruption around the site of the damaged muscle fibers (Järvinen et al., 2005). Immediate application of ice to a muscle tear or a contusion is standard treatment, with the goal being to reduce the proliferation of tissue degradation at the site of the injury (Järvinen et al., 2005). Evidence from animal models provides a strong rationale for the immediate post-injury application of cryotherapy to reduce the hematoma and inflammatory response, resulting in earlier regeneration of the injured tissue (Merrick et al., 1999; Deal et al., 2002; Schaser et al., 2006, 2007; Bleakley and Hopkins, 2010; Puntel et al., 2011). Further, evidence strongly supports the application of cryotherapy in the immediate stage following injury, as cryotherapy administered within the first 30 min of injury is a good window of opportunity for acute injury management (Merrick and McBrier, 2010). Surprisingly, although the clinical guidelines for post-injury ice application suggest application as soon as possible, several times a day for 15–20 min throughout the 72-h recovery period; (Michlovitz, 1990), there is no good clinical evidence in humans to support immediate icing or the clinically recommended rate of treatment. However, data from animal studies provide a strong rationale for immediate post-injury icing to reduce the hematoma and inflammation, resulting in earlier regeneration (Meeusen and Lievens, 1986; Hurme et al., 1993; Deal et al., 2002; Bleakley et al., 2004; Hubbard and Denegar, 2004).

The efficacy of both post-injury and post-exercise cryotherapy could be improved by delivering prolonged cooling to the damaged tissue within the first 24 h. To maximize efficacy, athletes and practitioners might opt to combine cryotherapy treatments. In practice, athletes could receive a standard 20–30 min ice treatment in the athletic training room or complete a CWI treatment in order to rapidly reduce both their intramuscular and core temperature, and follow this with the immediate application of PCM cooling packs over the muscle groups they wish to keep cool. Collectively, this approach will rapidly cool the tissue using more traditional techniques and would subsequently maintain the reduction of both peripheral and central temperatures for extended periods by administering 15°C PCM. This could allow the athlete to sustain the treatment effect from the acute cryotherapy for a longer duration in the immediate post-injury or post-exercise period and have a better chance to accelerate the recovery processes. The advantage of this approach is that the athlete can return to normal post-exercise activities (e.g., meal, relaxation, recreational activities) while receiving a cryotherapy dose with no risk of cold injury to the skin or other tissues.

Nevertheless, it would be remiss not to mention the growing trend throughout the literature recommending against icing injuries so as not to delay or impair the regeneration process or the natural healing response (Takagi et al., 2011; White and Wells, 2013). Most recently, the pioneer of the original rest, ice, compression, elevation (RICE) method originally proposed in 1978 (Mirkin and Hoffman, 1978), rescinded his recommendation of the practice because it may delay healing, instead of facilitating it (Mirkin, 2015). Mirkin proposed that the healing process requires an inflammatory response, which is impaired by the application of ice. Specifically, during the inflammatory phase of regeneration, the activity of insulin-like growth factor (IGF-1) is upregulated as it moves into the damaged tissues (Shi and Garry, 2006; Yin et al., 2013). Research in animal models has demonstrated that cryotherapy decreased IGF-1 expression (Takagi et al., 2011; Vieira Ramos et al., 2016), which might be harmful for muscle regeneration. However, this effect has not been demonstrated in humans, and the degree of muscle cooling occurring in animal models is substantially greater than that which occurs in humans (even over the course of 3 h of continuous PCM cooling; Kwiecien et al., 2019). There is no evidence in humans to support the notion that cryotherapy could delay recovery following both injury or exercise, and data from animal models should not be directly compared to what would happen in humans. There is some evidence suggesting that the therapeutic effects attributed to cryotherapy treatment might be due to a placebo effect (Broatch et al., 2014). This limitation is inherent in all cryotherapy-based research, due to the inability to rule out that beneficial results could be due to the participants preconceived belief about how cold exposure might benefit their recovery. However, it is unlikely that the placebo effect would explain in full the beneficial effect from prolonged PCM cooling on recovery of strength loss.

## Conclusion

The use of PCM cooling as a recovery modality offers a substitute to traditional cryotherapy modalities as it primarily allows for an increase in the duration of the cryotherapy dose in a safe way. The PCM cooling is well tolerated, safe, inexpensive, and delivers a prolonged cooling stimulus while allowing the athlete to continue with activities of daily living. Prolonging the duration of cryotherapy at physiologically comfortable temperatures might be the missing link for accelerating recovery through cryotherapy. Since CWI results in a more rapid initial decline in intramuscular temperature than PCM cooling (Kwiecien et al., 2019), a combination of CWI followed by PCM cooling could provide maximum cooling efficacy (rapid and prolonged). Future research should examine whether this combination further improves the recovery process and what the implications are for longer term athletic development. Future research should also establish evidence in support of prolonged cooling with 15°C PCM across various forms of exercise and whether a difference exists in the optimum duration of application. Gaining a greater understanding of the potential mechanisms by which 15°C PCM works for accelerating recovery will also advance the research, and potentially provide evidence in support of less noxious cooling in being considered a viable option as a treatment for recovery from exercise or soft tissue injury. On a similar note, establishing the potential benefits of prolonged cooling for acute injury management, post-operative inflammation and swelling and chronic disease such as rheumatological conditions, might lead to answers which could translate to the exercise recovery paradigm. Finally, understanding the implications on the adaptive responses to exercise training, particularly given the concerns over CWI and adaptation (for strength) would be of great value (Hyldahl and Peake, 2020).

## Author Contributions

All authors contributed to the drafting, editing, and approval of this mini-review.

## Conflict of Interest

The authors declare that the research was conducted in the absence of any commercial or financial relationships that could be construed as a potential conflict of interest.

## References

[B1] Allan R. Sharples A. P. Close G. L. Drust B. Shepherd S. O. Dutton J.. (2017). Postexercise cold water immersion modulates skeletal muscle PGC-1α mRNA expression in immersed and nonimmersed limbs: evidence of systemic regulation. J. Appl. Physiol. 123, 451–459. 10.1152/japplphysiol.00096.2017 28546467

[B2] Arbogast S. Reid M. B. (2004). Oxidant activity in skeletal muscle fibers is influenced by temperature, CO_2_ level, and muscle-derived nitric oxide. Am. J. Physiol. Regul. Integr. Comp. Physiol. 287, R698–R705. 10.1152/ajpregu.00072.2004 15178539

[B3] Armstrong R. B. (1990). Initial events in exercise-induced muscular injury. Med. Sci. Sports Exerc. 22, 429–435. 10.1249/00005768-199008000-00002 2205778

[B4] Armstrong R. B. Warren G. L. Warren J. A. (1991). Mechanisms of exercise-induced muscle fibre injury. Sports Med. 12, 184–207. 10.2165/00007256-199112030-00004 1784873

[B5] Bailey D. M. Erith S. J. Griffin P. J. Dowson A. Brewer D. S. Gant N.. (2007). Influence of cold-water immersion on indices of muscle damage following prolonged intermittent shuttle running. J. Sports Sci. 25, 1163–1170. 10.1080/02640410600982659 17654228

[B6] Banfi G. Lombardi G. Colombini A. Melegati G. (2010). Whole-body cryotherapy in athletes. Sports Med. 40, 509–517. 10.2165/11531940-000000000-00000 20524715

[B7] Banfi G. Melegati G. Barassi A. Dogliotti G. Melzi d'Eril G. Dugué B. (2009). Effects of whole-body cryotherapy on serum mediators of inflammation and serum muscle enzymes in athletes. J. Therm. Biol. 34, 55–59. 10.1016/j.jtherbio.2008.10.003

[B8] Barnett A. (2006). Using recovery modalities between training sessions in elite athletes. Sports Med. 36, 781–796. 10.2165/00007256-200636090-00005 16937953

[B9] Barwood M. J. Davey S. House J. R. Tipton M. J. (2009). Post-exercise cooling techniques in hot, humid conditions. Eur. J. Appl. Physiol. 107:385. 10.1007/s00421-009-1135-1 19649650

[B10] Bennett B. L. Hagan R. D. Huey K. A. Minson C. Cain D. (1995). Comparison of two cool vests on heat-strain reduction while wearing a firefighting ensemble. Eur. J. Appl. Physiol. Occup. Physiol. 70, 322–328. 10.1007/BF00865029 7649143

[B11] Bleakley C. M. Bieuzen F. Davison G. W. Costello J. T. (2014). Whole-body cryotherapy: empirical evidence and theoretical perspectives. Open Access J. Sports Med. 5, 25–36. 10.2147/OAJSM.S41655 24648779 PMC3956737

[B12] Bleakley C. M. Hopkins J. T. (2010). Is it possible to achieve optimal levels of tissue cooling in cryotherapy? Phys. Ther. Rev. 15, 344–350. 10.1179/174328810X12786297204873

[B13] Bleakley C. M. McDonough S. Gardner E. Baxter G. D. Hopkins J. T. Davison G. W. (2012). Cold-water immersion (cryotherapy) for preventing and treating muscle soreness after exercise. Cochrane Database System. Rev. 2:CD008262 10.1002/14651858.CD008262.pub2 PMC6492480 22336838

[B15] Bleakley C. M. McDonough S. MacAuley D. (2004). The use of ice in the treatment of acute soft-tissue injury. Am. J. Sports Med. 32, 251–261. 10.1177/0363546503260757 14754753

[B16] Brade C. Dawson B. Wallman K. Polglaze T. (2010). Postexercise cooling rates in 2 cooling jackets. J. Athletic Train. 45, 164–169. 10.4085/1062-6050-45.2.164 20210620 PMC2838468

[B17] Broatch J. R. Petersen A. Bishop D. J. (2014). Postexercise cold water immersion benefits are not greater than the placebo effect. Med. Sci. Sports Exerc. 46, 2139–2147. 10.1249/MSS.0000000000000348 24674975

[B18] Broatch J. R. Poignard M. Hausswirth C. Bishop D. J. Bieuzen F. (2019). Whole-body cryotherapy does not augment adaptations to high-intensity interval training. Sci. Rep. 9, 1–11. 10.1038/s41598-019-48518-1 31427654 PMC6700067

[B19] Brophy-Williams N. Landers G. Wallman K. (2011). Effect of immediate and delayed cold water immersion after a high intensity exercise session on subsequent run performance. J. Sports Sci. Med. 10, 665–670. 10.1016/j.jsams.2011.11.238 24149556 PMC3761518

[B20] Brownstein C. G. Ansdell P. Škarabot J. McHugh M. P. Howatson G. Goodall S.. (2019). The effect of phase change material on recovery of neuromuscular function following competitive soccer match-play. Front. Physiol. 10:647. 10.3389/fphys.2019.00647 31244667 PMC6562676

[B21] Cheng A. J. Willis S. J. Zinner C. Chaillou T. Ivarsson N. Ørtenblad N.. (2017). Post-exercise recovery of contractile function and endurance in humans and mice is accelerated by heating and slowed by cooling skeletal muscle. J. Physiol. 595, 7413–7426. 10.1113/JP274870 28980321 PMC5730848

[B22] Chou C. Tochihara Y. Kim T. (2008). Physiological and subjective responses to cooling devices on firefighting protective clothing. Eur. J. Appl. Physiol. 104, 369–374. 10.1007/s00421-007-0665-7 18259772

[B23] Ciolek J. J. (1985). Cryotherapy. Review of physiological effects and clinical application. Cleveland Clinic Q. 52, 193–201. 10.3949/ccjm.52.2.193 3896574

[B24] Clanton T. L. (2007). Hypoxia-induced reactive oxygen species formation in skeletal muscle. J. Appl. Physiol. 102, 2379–2388. 10.1152/japplphysiol.01298.2006 17289907

[B25] Clarkson P. M. Sayers S. P. (1999). Etiology of exercise-induced muscle damage. Can. J. Appl. Physiol. 24, 234–248. 10.1139/h99-020 10364418

[B26] Clifford T. Abbott W. Kwiecien S. Y. Howatson G. McHugh M. P. (2018). Cryotherapy reinvented: application of phase change material for recovery in elite soccer. Intern. J. Sports Physiol. Perform. 13, 584–589. 10.1123/ijspp.2017-0334 28872368

[B27] Costello J. T. Algar L. A. Donnelly A. E. (2011). Effects of whole-body cryotherapy (−110 °C) on proprioception and indices of muscle damage. Scand.J. Med. Sci. Sports 22, 190–198. 10.1111/j.1600-0838.2011.01292.x 21477164

[B28] Costello J. T. Baker P. R. A. Minett G. M. Bieuzen F. Stewart I. B. Bleakley C. (2015). Whole-body cryotherapy (extreme cold air exposure) for preventing and treating muscle soreness after exercise in adults. Cochrane Database System. Rev. 9:CD010789. 10.1002/14651858.CD010789.pub2 26383887 PMC9579836

[B29] Deal D. N. Tipton J. Rosencrance E. Curl W. W. Smith T. L. (2002). Ice reduces edema. J. Bone Joint Surgery-Ame. 84, 1573–1578. 10.2106/00004623-200209000-00009 12208913

[B30] Deschenes M. R. Kraemer W. J. Bush J. A. Doughty T. A. Kim D. Mullen K. M.. (1998). Biorhythmic influences on functional capacity of human muscle and physiological responses. Med. Sci. Sports Exerc. 30, 1399–1407. 10.1249/00005768-199809000-00008 9741608

[B31] Duffield R. Marino F. E. (2007). Effects of pre-cooling procedures on intermittent-sprint exercise performance in warm conditions. Eur. J. Appl. Physiol. 100, 727–735. 10.1007/s00421-007-0468-x 17476523

[B32] Dupuy O. Douzi W. Theurot D. Bosquet L. Dugu,é B. (2018). An evidence-based approach for choosing post-exercise recovery techniques to reduce markers of muscle damage, soreness, fatigue, and inflammation: a systematic review with meta-analysis. Front. Physiol. 9:403. 10.3389/fphys.2018.00403 29755363 PMC5932411

[B33] Dykstra J. H. Hill H. M. Miller M. G. Cheatham C. C. Michael T. J. Baker R. J. (2009). Comparisons of cubed ice, crushed ice, and wetted ice on intramuscular and surface temperature changes. J. Athletic Train. 44, 136–141. 10.4085/1062-6050-44.2.136 19295957 PMC2657028

[B34] Elias G. P. Wyckelsma V. L. Varley M. C. McKenna M. J. Aughey R. J. (2013). Effectiveness of water immersion on postmatch recovery in elite professional footballers. Intern. J. Sports Physiol. Perform. 8, 243–253. 10.1123/ijspp.8.3.243 22954483

[B35] Eston R. Peters D. (1999). Effects of cold water immersion on the symptoms of exercise-induced muscle damage. J. Sports Sci. 17, 231–238. 10.1080/026404199366136 10362390

[B36] Ferreira-Junior J. B. Bottaro M. Vieira A. Siqueira A. F. Vieira C. A. Durigan J. L. Q.. (2015). One session of partial-body cryotherapy (-110 °C) improves muscle damage recovery. Scand. J. Med. Sci. Sports 25, e524–e530. 10.1111/sms.12353 25556301

[B37] Fonda B. Sarabon N. (2013). Effects of whole-body cryotherapy on recovery after hamstring damaging exercise: a crossover study. Scand. J. Med. Sci. Sports 23, e270–e278. 10.1111/sms.12074 23614691

[B38] Friden J. Lieber R. L. (2001). Eccentric exercise-induced injuries to contractile and cytoskeletal muscle fibre components. Acta Physiol. Scand. 171, 321–326. 10.1046/j.1365-201x.2001.00834.x 11412144

[B39] Friden J. A. N. Lieber R. L. (1992). Structural and mechanical basis of exercise-induced muscle injury. Med. Sci. Sports Exerc. 24, 521–530. 10.1249/00005768-199205000-00005 1569848

[B40] Fuhrman F. A. Fuhrman G. J. Farr D. A. Fail J. H. (1961). Relationship between tissue respiration and total metabolic rate in hypo- and normothermic rats. Am. J. Physiol. Legacy Content 201, 231–234. 10.1152/ajplegacy.1961.201.2.231 13702444

[B41] Fuhrman G. J. (1959). Oxygen consumption of animals and tissues as a function of temperature. J. General Physiol. 42, 715–722. 10.1085/jgp.42.4.715 13631198 PMC2195002

[B42] Gage A. A. (1979). Cryo corner: what temperature is lethal for cells? J. Dermatol. Surgery Oncol. 5, 459–464. 10.1111/j.1524-4725.1979.tb00695.x 110858

[B43] Gao C. Kuklane K. Holmer I. (2010). Cooling vests with phase change material packs: the effects of temperature gradient, mass and covering area. Ergonomics 53, 716–723. 10.1080/00140130903581649 20432090

[B44] Gao C. Kuklane K. Holmér I. (2011). Cooling vests with phase change materials: the effects of melting temperature on heat strain alleviation in an extremely hot environment. Eur. J. Appl. Physiol. 111, 1207–1216. 10.1007/s00421-010-1748-4 21127896

[B45] Garcia C. da Mota G. Marocolo M. (2016). Cold water immersion is acutely detrimental but increases performance post-12 h in rugby players. Intern. J. Sports Med. 37, 619–624. 10.1055/s-0035-1565200 27136509

[B46] Gregson W. Black M. A. Jones H. Milson J. Morton J. Dawson B.. (2011). Influence of cold water immersion on limb and cutaneous blood flow at rest. Am. J. Sports Med. 39, 1316–1323. 10.1177/0363546510395497 21335348

[B47] Guilhem G. Hug F. Couturier A. Regnault S. Bournat L. Filliard J.-R.. (2013). Effects of air-pulsed cryotherapy on neuromuscular recovery subsequent to exercise-induced muscle damage. Am. J. Sports Med. 41, 1942–1951. 10.1177/0363546513490648 23739686

[B48] Gulick D. T. Kimura I. F. Sitler M. Paolone A. Kelly J. D. (1996). Various treatment techniques on signs and symptoms of delayed onset muscle soreness. J. Athl. Train 31, 145–152. 16558388 PMC1318445

[B49] Halson S. L. Quod M. J. Martin D. T. Gardner A. S. Ebert T. R. Laursen P. B. (2008). Physiological responses to cold water immersion following cycling in the heat. Intern. J. Sports Physiol. Perform. 3, 331–346. 10.1123/ijspp.3.3.331 19211945

[B50] Hassabo A. G. (2014). New approaches to improving thermal regulating property of cellulosic fabric. Carbohydrate Polymers 101, 912–919. 10.1016/j.carbpol.2013.10.006 24299856

[B51] Hausswirth C. Duffield R. Pournot H. Bieuzen F. Louis J. Brisswalter J.. (2012). Postexercise cooling interventions and the effects on exercise-induced heat stress in a temperate environment. Appl. Physiol. Nutrition Metabol. 37, 965–975. 10.1139/h2012-077 22827512

[B52] Hausswirth C. Le Meur Y. (2011). Physiological and nutritional aspects of post-exercise recovery. Sports Med. 41, 861–882. 10.2165/11593180-000000000-00000 21923203

[B53] Hausswirth C. Louis J. Bieuzen F. Pournot H. Fournier J. Filliard J.-R.. (2011). Effects of whole-body cryotherapy vs. far-infrared vs. passive modalities on recovery from exercise-induced muscle damage in highly-trained runners. PLoS ONE 6:e27749. 10.1371/journal.pone.0027749 22163272 PMC3233540

[B54] Heyman E. De Geus B. A. S. Mertens I. Meeusen R. (2009). Effects of four recovery methods on repeated maximal rock climbing performance. Med. Sci. Sports Exerc. 41, 1303–1310. 10.1249/MSS.0b013e318195107d 19461534

[B55] Ho S. S. Illgen R. L. Meyer R. W. Torok P. J. Cooper M. D. Reider B. (1995). Comparison of various icing times in decreasing bone metabolism and blood flow in the knee. Am. J. Sports Med. 23, 74–76. 10.1177/036354659502300112 7726354

[B56] Hohenauer E. Taeymans J. Baeyens J.-P. Clarys P. Clijsen R. (2015). The effect of post-exercise cryotherapy on recovery characteristics: a systematic review and meta-analysis. PLoS ONE 10:e0139028. 10.1371/journal.pone.0139028 26413718 PMC4586380

[B57] House J. R. Lunt H. C. Taylor R. Milligan G. Lyons J. A. House C. M. (2013). The impact of a phase-change cooling vest on heat strain and the effect of different cooling pack melting temperatures. Eur. J. Appl. Physiol. 113, 1223–1231. 10.1007/s00421-012-2534-2 23160652

[B58] Howatson G. van Someren K. A. (2008). The prevention and treatment of exercise-induced muscle damage. Sports Med. 38, 483–503. 10.2165/00007256-200838060-00004 18489195

[B59] Hubbard T. J. Denegar C. R. (2004). Does cryotherapy improve outcomes with soft tissue injury? J. Athl. Train 39, 278–279. 15496998 PMC522152

[B60] Hurme T. Rantanen J. Kaliomo H. (1993). Effects of early cryotherapy in experimental skeletal muscle injury. Scand. J. Med. Sci. Sports 3, 46–51. 10.1111/j.1600-0838.1993.tb00360.x

[B61] Hyldahl R. D. Peake J. M. (2020). Combining cooling or heating applications with exercise training to enhance performance and muscle adaptations. J. Appl. Physiol. 129, 353–365. 10.1152/japplphysiol.00322.2020 32644914

[B62] Ihsan M. Abbiss C. R. Gregson W. Allan R. (2020). Warming to the Ice Bath: Don't Go Cool on Cold Water Immersion Just Yet! Temperature. 10.1080/23328940.2020.1727085 PMC7577239 33134431

[B63] Ihsan M. Watson G. Abbiss C. R. (2016). What are the physiological mechanisms for post-exercise cold water immersion in the recovery from prolonged endurance and intermittent exercise? Sports Med. 46, 1095–1109. 10.1007/s40279-016-0483-3 26888646

[B64] Ihsan M. Watson G. Lipski M. Abbiss C. R. (2013). Influence of postexercise cooling on muscle oxygenation and blood volume changes. Med. Sci. Sports Exercise 45, 876–882. 10.1249/MSS.0b013e31827e13a2 23247707

[B65] Ingram J. Dawson B. Goodman C. Wallman K. Beilby J. (2009). Effect of water immersion methods on post-exercise recovery from simulated team sport exercise. J. Sci. Med. Sport 12, 417–421. 10.1016/j.jsams.2007.12.011 18547863

[B66] Järvinen T. A. H. Järvinen T. L. N. Kääriäinen M. Kalimo H. Järvinen M. (2005). Muscle injuries: biology and treatment. Am. J. Sports Med. 33, 745–764. 10.1177/0363546505274714 15851777

[B67] Kendall B. Eston R. (2002). Exercise-induced muscle damage and the potential protective role of estrogen. Sports Med. 32, 103–123. 10.2165/00007256-200232020-00003 11817996

[B68] Kenny G. P. Schissler A. R. Stapleton J. Piamonte M. Binder K. Lynn A.. (2011). Ice cooling vest on tolerance for exercise under uncompensable heat stress. J. Occup. Environ. Hygiene 8, 484–491. 10.1080/15459624.2011.596043 21756138

[B69] Knight K. L. (1985). Cryotherapy: Theory, Technique, and Physiology. Chattanooga, TN: Chattanooga Corp., Education Division.

[B70] Knight K. L. (1995). Cryotherapy in Sport Injury Management. Champaign: Human Kinetics.

[B71] Krueger M. Costello J. T. Achtzehn S. Dittmar K.-H. Mester J. (2019). Whole-body cryotherapy (−110°C) following high-intensity intermittent exercise does not alter hormonal, inflammatory or muscle damage biomarkers in trained males. Cytokine 113, 277–284. 10.1016/j.cyto.2018.07.018 30031682

[B72] Kwiecien S. Y. McHugh M. P. Goodall S. Hicks K. M. Hunter A. M. Howatson G. (2019). Exploring the efficacy of a safe cryotherapy alternative: physiological temperature changes from cold-water immersion versus prolonged cooling of phase-change material. Intern. J. Sports Physiol. Perform. 14, 1–26. 10.1123/ijspp.2018-0763 30958051

[B73] Kwiecien S. Y. McHugh M. P. Hicks K. M. Keane K. M. Howatson G. (2020b). The efficacy of prolonged cooling using phase change material for enhancing recovery following a marathon: 2367: Board# 16 May 27 9:30 AM-11:00 AM. Med. Sci. Sports Exerc. 52:5(Supplement). 10.1080/02640414.2017.1312492

[B74] Kwiecien S. Y. McHugh M. P. Howatson G. (2018). The efficacy of cooling with phase change material for the treatment of exercise-induced muscle damage: pilot study. J. Sports Sci. 36, 407–413. 28391765 10.1080/02640414.2017.1312492

[B75] Kwiecien S. Y. O'Hara D. J. McHugh M. P. Howatson G. (2020a). Prolonged cooling with phase change material enhances recovery and does not affect the subsequent repeated bout effect following exercise. Eur. J. Appl. Physiol. 120, 413–423. 10.1007/s00421-019-04285-5 31828479

[B76] Lane K. N. Wenger H. A. (2004). Effect of selected recovery conditions on performance of repeated bouts of intermittent cycling separated by 24 hours. J. Strength Cond. Res. 18:855. 10.1519/14183.1 15574106

[B77] Lapointe B. M. Frenette J. Côté C. H. (2002). Lengthening contraction-induced inflammation is linked to secondary damage but devoid of neutrophil invasion. J. Appl. Physiol. 92, 1995–2004. 10.1152/japplphysiol.00803.2001 11960950

[B78] Leeder J. Gissane C. van Someren K. Gregson W. Howatson G. (2012). Cold water immersion and recovery from strenuous exercise: a meta-analysis. Br. J. Sports Med. 46, 233–240. 10.1136/bjsports-2011-090061 21947816

[B79] Leeder J. D. C. Godfrey M. Gibbon D. Gaze D. Davison G. W. van Someren K. A.. (2019). Cold water immersion improves recovery of sprint speed following a simulated tournament. Eur. J. Sport Sci. 19, 1166-1174. 10.1080/17461391.2019.1585478 30957673

[B80] Lieber R. L. (2018). Biomechanical response of skeletal muscle to eccentric contractions. J. Sport Health Sci. 7, 294–309. 10.1016/j.jshs.2018.06.005 30356666 PMC6189273

[B81] Lombardi G. Ziemann E. Banfi G. (2017). Whole-body cryotherapy in athletes: from therapy to stimulation. An updated review of the literature. Front. Physiol. 8:258. 10.3389/fphys.2017.00258 28512432 PMC5411446

[B82] Mac Auley D. C. (2001). Ice therapy: how good is the evidence? Intern. J. Sports Med. 22, 379–384. 10.1055/s-2001-15656 11510876

[B83] Machado A. F. Almeida A. C. Micheletti J. K. Vanderlei F. M. Tribst M. F. Netto Junior J.. (2016). Dosages of cold-water immersion post exercise on functional and clinical responses: a randomized controlled trial. Scand. J. Med. Sci. Sports 27, 1356–1363. 10.1111/sms.12734 27430594

[B84] Machado A. F. Ferreira P. H. Micheletti J. K. de Almeida A. C. Lemes Í. R. Vanderlei F. M.. (2015). Can water temperature and immersion time influence the effect of cold water immersion on muscle soreness? A systematic review and meta-analysis. Sports Med. 46, 503–514. 10.1007/s40279-015-0431-7 26581833 PMC4802003

[B85] Mawhinney C. Heinonen I. Low D. A. Han C. Jones H. Kalliokoski K. K.. (2020). Changes in quadriceps femoris muscle perfusion following different degrees of cold-water immersion. J. Appl. Physiol. Bethesda, Md. : 1985. 10.1152/japplphysiol.00833.2019 32352343

[B86] Mawhinney C. Jones H. Joo C. H. Low D. A. Green D. J. Gregson W. (2013). Influence of cold-water immersion on limb and cutaneous blood flow after exercise. Med. Sci. Sports Exerc. 45, 2277–2285. 10.1249/MSS.0b013e31829d8e2e 24240118

[B87] McHugh M. P. Clifford T. Abbott W. Kwiecien S. Y. Kremenic I. J. DeVita J. J.. (2019). Countermovement jump recovery in professional soccer players using an inertial sensor. Intern. J. Sports Physiol. Perform. 14, 9–15. 10.1123/ijspp.2018-0131 29809065

[B88] Meeusen R. Lievens P. (1986). The use of cryotherapy in sports injuries. Sports Med. 3, 398–414. 10.2165/00007256-198603060-00002 3538270

[B89] Merrick M. A. (2002). Secondary injury after musculoskeletal trauma: a review and update. J. Athl. Train 37, 209–217. 16558673 PMC164347

[B90] Merrick M. A. Jutte L. S. Smith M. E. (2003). Cold modalities with different thermodynamic properties produce different surface and intramuscular temperatures. J. Athl. Train 38, 28–33. 12937469 PMC155508

[B91] Merrick M. A. McBrier N. M. (2010). Progression of secondary injury after musculoskeletal trauma—a window of opportunity? J. Sport Rehabil. 19, 380–388. 10.1123/jsr.19.4.380 21116007

[B92] Merrick M. A. Rankin J. M. Andres F. A. Hinman C. L. (1999). A preliminary examination of cryotherapy and secondary injury in skeletal muscle. Med. Sci. Sports Exerc. 31:1516. 10.1097/00005768-199911000-00004 10589851

[B93] Michlovitz S. L. (1990). Thermal Agents in Rehabilitation. Davis, CA: F. A. Davis Company.

[B94] Minett G. M. Duffield R. (2014). Is recovery driven by central or peripheral factors? A role for the brain in recovery following intermittent-sprint exercise. Front. Physiol. 5:24. 10.3389/fphys.2014.00024 24550837 PMC3909945

[B95] Minett G. M. Duffield R. Billaut F. Cannon J. Portus M. R. Marino F. E. (2014). Cold-water immersion decreases cerebral oxygenation but improves recovery after intermittent-sprint exercise in the heat. Scand. J. Med. Sci. Sports 24, 656–666. 10.1111/sms.12060 23458430

[B96] Minett G. M. Duffield R. Kellett A. Portus M. (2012). Effects of mixed-method cooling on recovery of medium-fast bowling performance in hot conditions on consecutive days. J. Sports Sci. 30, 1387–1396. 10.1080/02640414.2012.709267 22867101

[B97] Mirkin G. (2015). Why Ice Delays Recovery. Available online at https://www.drmirkin.com/fitness/why-ice-delays-recovery.html (accessed June 10, 2020).

[B98] Mirkin G. Hoffman M. (1978). The Sports Medicine Book. Boston, MA: Little Brown.

[B99] Montgomery P. G. Pyne D. B. Hopkins W. G. Dorman J. C. Cook K. Minahan C. L. (2008). The effect of recovery strategies on physical performance and cumulative fatigue in competitive basketball. J. Sports Sci. 26, 1135–1145. 10.1080/02640410802104912 18608847

[B100] Mortensen S. P. Damsgaard R. Dawson E. A. Secher N. H. González-Alonso J. (2008). Restrictions in systemic and locomotor skeletal muscle perfusion, oxygen supply and VO2 during high-intensity whole-body exercise in humans. J. Physiol. (Lond). 586, 2621–2635. 10.1113/jphysiol.2007.149401 18372307 PMC2464345

[B101] Mullaney M. J. McHugh M. P. Kwiecien S. Y. Ioviero N. Fink A. Howatson G. (2020). Accelerated recovery of muscle function in baseball pitchers using post-game phase change material cooling. 2020 Comb. Sect. Meeting. 10.1249/MSS.0000000000002447. [Epub ahead of print]. 32694373

[B102] Nogueira N. M. Felappi C. J. Lima C. S. Medeiros D. M. (2019). Effects of local cryotherapy for recovery of delayed onset muscle soreness and strength following exercise-induced muscle damage: systematic review and meta-analysis. Sport Sci. Health 16, 1–11. 10.1007/s11332-019-00571-z

[B103] Oakley E. T. Pardeiro R. B. Powell J. W. Millar A. L. (2013). The Effects of Multiple Daily applications of ice to the hamstrings on biochemical measures, signs, and symptoms associated with exercise-induced muscle damage. J. Strength Cond. Res. 27, 2743–2751. 10.1519/JSC.0b013e31828830df 23364294

[B104] Osterman A. L. Heppenstall R. B. Sapega A. A. Katz M. Chance B. Sokolow D. (1984). Muscle Ischemia and Hypothermia: a bioenergetic study using 31phosphorus nuclear magnetic resonance spectroscopy. J. Trauma 24, 811–817. 10.1097/00005373-198409000-00006 6481831

[B105] Peiffer J. J. Abbiss C. R. Watson G. Nosaka K. Laursen P. B. (2008). Effect of a 5-min cold-water immersion recovery on exercise performance in the heat. Br. J. Sports Med. 44, 461–465. 10.1136/bjsm.2008.048173 18539654

[B106] Peiffer J. J. Abbiss C. R. Watson G. Nosaka K. Laursen P. B. (2009). Effect of cold-water immersion duration on body temperature and muscle function. J. Sports Sci. 27, 987–993. 10.1080/02640410903207424 19847682

[B107] Pointon M. Duffield R. (2012). Cold water immersion recovery after simulated collision sport exercise. Med. Sci. Sports Exerc. 44, 206–216. 10.1249/MSS.0b013e31822b0977 21716151

[B108] Pointon M. Duffield R. Cannon J. Marino F. E. (2011). Cold water immersion recovery following intermittent-sprint exercise in the heat. Eur. J. Appl. Physiol. 112, 2483–2494. 10.1007/s00421-011-2218-3 22057508

[B109] Poppendieck W. Faude O. Wegmann M. Meyer T. (2013). Cooling and performance recovery of trained athletes: a meta-analytical review. Intern. J. Sports Physiol. Perform. 8, 227–242. 10.1123/ijspp.8.3.227 23434565

[B110] Pournot H. Bieuzen F. Duffield R. Lepretre P. M. Cozzolino C. Hausswirth C. (2010). Short term effects of various water immersions on recovery from exhaustive intermittent exercise. Eur. J. Appl. Physiol. 111, 1287–1295. 10.1007/s00421-010-1754-6 21132438

[B111] Pournot H. Bieuzen F. Louis J. Fillard J. R. Barbiche E. Hausswirth C. (2011). Time-course of changes in inflammatory response after whole-body cryotherapy multi exposures following severe exercise. PLoS ONE 6:e22748. 10.1371/journal.pone.0022748 21829501 PMC3145670

[B112] Proske U. Allen T. J. (2005). Damage to skeletal muscle from eccentric exercise. Exerc. Sport Sci. Rev. 33, 98–104. 10.1097/00003677-200504000-00007 15821431

[B113] Proske U. Morgan D. L. (2001). Muscle damage from eccentric exercise: mechanism, mechanical signs, adaptation and clinical applications. J. Physiol. 537, 333–345. 10.1111/j.1469-7793.2001.00333.x 11731568 PMC2278966

[B114] Puntel G. O. Carvalho N. R. Amaral G. P. Lobato L. D. Silveira S. O. Daubermann M. F.. (2011). Therapeutic cold: an effective kind to modulate the oxidative damage resulting of a skeletal muscle contusion. Free Rad. Res. 45, 125–138. 10.3109/10715762.2010.517252 20942569

[B115] Purvis A. J. Cable N. T. (2000). The effects of phase control materials on hand skin temperature within gloves of soccer goalkeepers. Ergonomics 43, 1480–1488. 10.1080/001401300750003916 11083129

[B116] Roberts L. A. Muthalib M. Stanley J. Lichtwark G. Nosaka K. Coombes J. S.. (2015). Effects of cold water immersion and active recovery on hemodynamics and recovery of muscle strength following resistance exercise. Am. J. Physiol. Regul. Integr. Comp. Physiol. 309, R389–R398. 10.1152/ajpregu.00151.2015 26062633

[B117] Rose C. Edwards K. M. Siegler J. Graham K. Caillaud C. (2017). Whole-body cryotherapy as a recovery technique after exercise: a review of the literature. Int. J. Sports Med. 38, 1049–1060. 10.1055/s-0043-114861 29161748

[B118] Rowsell G. J. Coutts A. J. Reaburn P. Hill-Haas S. (2009). Effects of cold-water immersion on physical performance between successive matches in high-performance junior male soccer players. J. Sports Sci. 27, 565–573. 10.1080/02640410802603855 19308790

[B119] Rowsell G. J. Coutts A. J. Reaburn P. Hill-Haas S. (2011). Effect of post-match cold-water immersion on subsequent match running performance in junior soccer players during tournament play. J. Sports Sci. 29, 1–6. 10.1080/02640414.2010.512640 21077001

[B120] Sapega A. A. Heppenstall R. B. Sokolow D. P. Graham T. J. Maris J. M. Ghosh A. K.. (1988). The bioenergetics of preservation of limbs before replantation. The rationale for intermediate hypothermia. J. Bone Joint Surgery 70, 1500–1513. 10.2106/00004623-198870100-00010 3198676

[B121] Schaal K. Le Meur Y. Louis J. Filliard J.-R. Hellard P. Casazza G.. (2015). Whole-body cryostimulation limits overreaching in elite synchronized swimmers. Med. Sci. Sports Exerc. 47, 1416–1425. 10.1249/MSS.0000000000000546 25314578

[B122] Schaser K. D. Disch A. C. Stover J. F. Lauffer A. Bail H. J. Mittlmeier T. (2007). Prolonged superficial local cryotherapy attenuates microcirculatory impairment, regional inflammation, and muscle necrosis after closed soft tissue injury in rats. Am. J. Sports Med. 35, 93–102. 10.1177/0363546506294569 17197574

[B123] Schaser K. D. Stover J. F. Melcher I. Lauffer A. Haas N. P. Bail H. J.. (2006). Local cooling restores microcirculatory hemodynamics after closed soft-tissue trauma in rats. J. Trauma 61, 642–649. 10.1097/01.ta.0000174922.08781.2f 16967001

[B124] Selfe J. Hardaker N. Whitaker J. Hayes C. (2007). Thermal imaging of an ice burn over the patella following clinically relevant cryotherapy application during a clinical research study. Phys. Ther. Sport 8, 153–158. 10.1016/j.ptsp.2007.04.001

[B125] Shi X. Garry D. J. (2006). Muscle stem cells in development, regeneration, and disease. Genes Dev. 20, 1692–1708. 10.1101/gad.1419406 16818602

[B126] Siqueira A. F. Vieira A. Bottaro M. Ferreira-Júnior J. B. Nóbrega O. de T.. (2018). Multiple cold-water immersions attenuate muscle damage but not alter systemic inflammation and muscle function recovery: a parallel randomized controlled trial. Sci. Rep. 8:10961. 10.1038/s41598-018-28942-5 30026562 PMC6053395

[B127] Siqueira A. F. Vieira A. Ramos G. V. de Cássia Marqueti R. de Fátima Salvini T. Puntel G. O.. (2016). Multiple cryotherapy applications attenuate oxidative stress following skeletal muscle injury. Redox Report 22, 323–329. 10.1080/13510002.2016.1239880 27750503 PMC6837703

[B128] Spiteller G. (2006). Peroxyl radicals: Inductors of neurodegenerative and other inflammatory diseases. Their origin and how they transform cholesterol, phospholipids, plasmalogens, polyunsaturated fatty acids, sugars, and proteins into deleterious products. Free Rad. Biol. Med. 41, 362–387. 10.1016/j.freeradbiomed.2006.03.013 16843819

[B129] Staublr W. T. (1989). Eccentric action of muscles. Exerc. Sport Sci. Rev. 16, 157–186. 10.1249/00003677-198900170-00008 2676546

[B130] Stephens J. M. Sharpe K. Gore C. Miller J. Slater G. J. Versey N.. (2018). Core temperature responses to cold-water immersion recovery: a pooled-data analysis. Intern. J. Sports Physiol. Perform. 13, 917–925. 10.1123/ijspp.2017-0661 29283744

[B131] Supinski G. S. Callahan L. A. (2007). Free radical-mediated skeletal muscle dysfunction in inflammatory conditions. J. Appl. Physiol. 102, 2056–2063. 10.1152/japplphysiol.01138.2006 17218425

[B132] Swenson C. Swärd L. Karlsson J. (2007). Cryotherapy in sports medicine. Scand. J. Med. Sci. Sports 6, 193–200. 10.1111/j.1600-0838.1996.tb00090.x 8896090

[B133] Takagi R. Fujita N. Arakawa T. Kawada S. Ishii N. Miki A. (2011). Influence of icing on muscle regeneration after crush injury to skeletal muscles in rats. J. Appl. Physiol. 110, 382–388. 10.1152/japplphysiol.01187.2010 21164157

[B134] Tate M. Forster D. Mainwaring D. E. (2008). Influence of garment design on elite athlete cooling. Sports Technol. 1, 117–124. 10.1080/19346182.2008.9648462

[B135] Tee J. C. Bosch A. N. Lambert M. I. (2007). Metabolic consequences of exercise-induced muscle damage. Sports Med. 37, 827–836. 10.2165/00007256-200737100-00001 17887809

[B136] Tiest W. M. B. Kosters N. D. Kappers A. M. L. Daanen H. A. M. (2012). Phase change materials and the perception of wetness. Ergonomics 55, 508–512. 10.1080/00140139.2011.645886 22423680

[B137] Tipton M. J. Collier N. Massey H. Corbett J. Harper M. (2017). Cold water immersion: kill or cure? Experim. Physiol. 102, 1335–1355. 10.1113/EP086283 28833689

[B138] Vaile J. Halson S. Gill N. Dawson B. (2007). Effect of hydrotherapy on the signs and symptoms of delayed onset muscle soreness. Eur. J. Appl. Physiol. 102, 447–455. 10.1007/s00421-007-0605-6 17978833

[B139] Vaile J. Halson S. Gill N. Dawson B. (2008). Effect of cold water immersion on repeat cycling performance and thermoregulation in the heat. J. Sports Sci. 26, 431–440. 10.1080/02640410701567425 18274940

[B140] Vaile J. O'Hagan C. Stefanovic B. Walker M. Gill N. Askew C. D. (2010). Effect of cold water immersion on repeated cycling performance and limb blood flow. Br. J. Sports Med. 45, 825–829. 10.1136/bjsm.2009.067272 20233843

[B141] Versey N. Halson S. Dawson B. (2011). Effect of contrast water therapy duration on recovery of cycling performance: a dose-response study. Eur. J. Appl. Physiol. 111, 37–46. 10.1007/s00421-010-1614-4 20809231

[B142] Versey N. G. Halson S. L. Dawson B. T. (2013). Water immersion recovery for athletes: effect on exercise performance and practical recommendations. Sports Med. 43, 1101–1130. 10.1007/s40279-013-0063-8 23743793

[B143] Vieira Ramos G. Pinheiro C. M. Messa S. P. Delfino G. B. Marqueti R. de C.. (2016). Cryotherapy reduces inflammatory response without altering muscle regeneration process and extracellular matrix remodeling of rat muscle. Sci. Rep. 6:18525. 10.1038/srep18525 26725948 PMC4698758

[B144] Vieira A. Bottaro M. Ferreira-Junior J. B. Vieira C. Cleto V. A. Cadore E. L.. (2015). Does whole-body cryotherapy improve vertical jump recovery following a high-intensity exercise bout? Open Access J. Sports Med. 6, 49–54. 10.2147/OAJSM.S70263 25750548 PMC4348140

[B145] Webb N. P. Harris N. K. Cronin J. B. Walker C. (2013). The relative efficacy of three recovery modalities after professional rugby league matches. J. Strength Cond. Res. 27, 2449–2455. 10.1519/JSC.0b013e31827f5253 23238097

[B146] White G. E. Wells G. D. (2013). Cold-water immersion and other forms of cryotherapy: physiological changes potentially affecting recovery from high-intensity exercise. Extreme Physiol. Med. 2:26. 10.1186/2046-7648-2-26 24004719 PMC3766664

[B147] Wilcock I. M. Cronin J. B. Hing W. A. (2006). Physiological response to water immersion. Sports Med. 36, 747–765. 10.2165/00007256-200636090-00003 16937951

[B148] Wilke B. Weiner R. D. (2003). Postoperative cryotherapy: risks versus benefits of continuous-flow cryotherapy units. Clin. Podiatric Med. Surgery 20, 307–322. 10.1016/S0891-8422(03)00009-0 12776983

[B149] Wilson L. J. Cockburn E. Paice K. Sinclair S. Faki T. Hills F. A.. (2018). Recovery following a marathon: a comparison of cold water immersion, whole body cryotherapy and a placebo control. Eur. J. Appl. Physiol. 118, 153–163. 10.1007/s00421-017-3757-z 29127510

[B150] Yackzan L. Adams C. Francis K. T. (1984). The effects of ice massage on delayed muscle soreness. Am. J. Sports Med. 12, 159–165. 10.1177/036354658401200214 6742292

[B151] Yeargin S. W. Casa D. J. McClung J. M. Knight J. C. Healey J. C. Goss P. J.. (2006). Body Cooling between two bouts of exercise in the heat enhances subsequent performance. J. Strength Cond. Res. 20:383. 10.1519/R-18075.1 16686568

[B152] Yin H. Price F. Rudnicki M. A. (2013). Satellite cells and the muscle stem cell niche. Physiol. Rev. 93, 23–67. 10.1152/physrev.00043.2011 23303905 PMC4073943

[B153] Zhang H. (2003). Human thermal sensation and comfort in transient and non-uniform thermal environment (Ph.D. thesis), University of California, Berkeley, Berkeley, CA, United States.

[B154] Ziemann E. Olek R. A. Kujach S. Grzywacz T. Antosiewicz J. Garsztka T.. (2012). Five-day whole-body cryostimulation, blood inflammatory markers, and performance in high-ranking professional tennis players. J. Athletic Training 47, 664–672. 10.4085/1062-6050-47.6.13 23182015 PMC3499891

